# Scopoletin Suppresses Activation of Dendritic Cells and Pathogenesis of Experimental Autoimmune Encephalomyelitis by Inhibiting NF-κB Signaling

**DOI:** 10.3389/fphar.2019.00863

**Published:** 2019-08-02

**Authors:** Fei Zhang, Yuan Zhang, Ting Yang, Ze-Qing Ye, Jing Tian, Hai-Rong Fang, Juan-Juan Han, Zhe-Zhi Wang, Xing Li

**Affiliations:** National Engineering Laboratory for Resource Development of Endangered Crude Drugs in Northwest China, The Key Laboratory of Medicinal Resources and Natural Pharmaceutical Chemistry, The Ministry of Education, College of Life Sciences, Shaanxi Normal University, Xi’an, China

**Keywords:** scopoletin, experimental autoimmune encephalomyelitis, multiple sclerosis, dendritic cells, NF-κB signaling

## Abstract

Scopoletin, a phenolic coumarin derived from many medical or edible plants, is involved in various pharmacological functions. In the present study, we showed that Scopoletin effectively ameliorated experimental autoimmune encephalomyelitis (EAE), an animal model of multiple sclerosis (MS), through novel regulatory mechanisms involving inhibition of NF-κB activity in dendritic cells (DCs). Scopoletin treatment significantly improved the severity of the disease and prominently decreased inflammation and demyelination of central nervous system (CNS) in EAE mice. Disease alleviation correlated with the downregulation of major histocompatibility complex (MHC) class II, CD80 and CD86, expressed on DCs of CNS or spleens, and the infiltration and polarization of encephalitogenic Th1/Th17 cells. Consistent with the *in vivo* data, Scopoletin-treated, bone marrow-derived dendritic cells (BM-DCs) exhibited reduced expression of MHC class II and costimulatory molecules (e.g., CD80 and CD86) and reduced NF-κB phosphorylation. These findings, for the first time, demonstrated the ability of Scopoletin to impair DC activation, downregulating pathogenic Th1/Th17 inflammatory cell responses and, eventually, reducing EAE severity. Our study demonstrates new evidence that natural products derived from medical or edible plants, such as Scopoletin, will be valuable in developing a novel therapeutic agent for MS in the future.

## Introduction

Multiple sclerosis (MS) is a T-cell-mediated chronic autoimmune disease featured by neuroinflammation and demyelination in the central nervous system (CNS), affecting 2.5 million people worldwide, and the incidence continues to increase (Hemmer et al., 2015). Experimental autoimmune encephalomyelitis (EAE) is a well-known animal model of MS that is used to study the underlying mechanism and that provides the theoretical basis for developing new therapies of MS (Procaccini et al., 2015; Lassmann and Bradl, 2016).

Dendritic cells (DCs) are antigen-presenting cells that connect the innate and adaptive immune response. Depending on the variety activation status, DCs can either activate or modulate naive T lymphocytes. When DCs are activated, costimulatory molecules are upregulated and produce cytokines that drive T-cell priming and effector differentiation (Amodio and Gregori, 2012). In the absence of activation, antigen presentation by steady-state DCs can result to T-cell unresponsiveness and tolerance (Besusso et al., 2015). Therefore, modulation of DCs has been shown to be a promising area for the treatment of autoimmune disease. For instance, it was proved that DCs treated with natural compounds or synthetic drugs blocked the inflammatory signal and halted disease development (Ginwala et al., 2016; Kornberg et al., 2018). DC-induced suppression is mainly dependent on inhibiting Th17 cell differentiation or promoting Treg cell generation (Thome et al., 2014a; Thome et al., 2018). It is known that naive CD4^+^ T cells can develop into various effector subsets containing Th1, Th2, Th17, and Treg cells, etc.; among these, Th17 cells are crucially related to the pathogenesis of MS/EAE (He et al., 2017; Malik et al., 2017). Suppression of the cellular response, Th17 cells in particular, can therefore act as a therapeutic target for controlling autoimmune and inflammatory diseases.

NF-κB is a broad transcription factor which can regulate various biological functions containing inflammation, immunity, cell growth, and survival, and its signaling pathway plays a role in DC activation and maturation (Ardeshna et al., 2000). Phosphorylation of NF-κB in innate immune cells induces inflammation reaction, cytokine secretion, and tissue damage, thus promoting to the progress of autoimmune diseases including EAE (Lu et al., 2013). In addition, immature DCs respond to danger signals or antigen and, when mature, produce abundance of proinflammatory cytokines, such as type I interferons (IFN). Subsequently, mature DCs present antigens to T lymphocytes to stimulate a sustained immune response, given the interferon regulatory factor (IRF) family’s important role in DC development and maturation. For example, IRF-7 is necessary for producing IFN-α and IFN-β, and it serves as the prime regulator of type-I interferon-dependent immune responses (Honda et al., 2005; Gabriele and Ozato, 2007; Yasuda et al., 2007).

Scopoletin is a phenolic coumarin extracted from many medical plants, including *Erycibe obtusifolia*, *Aster tataricus*, *Foeniculum vulgare*, and *Artemisia iwayomogi*, as well as some edible plants, such as *Lycium barbarum* and *Morinda citrifolia* (Dou et al., 2013; Lee et al., 2014; Forino et al., 2016; Shalan et al., 2016). Scopoletin has been shown to possess anti-inflammatory, antioxidant, antidepressant, hypouricemic, and neuroprotective effects (Ding et al., 2005; Basu et al., 2016). Moreover, a recent report suggests that Scopoletin is effective in an adjuvant-induced arthritis rat model, increasing the possibility of its application as a therapeutic agent for autoimmune diseases (Pan et al., 2010). However, little is known about the regulatory properties and underlying mechanism of Scopoletin relative to innate immune cells such as DCs and its effect on the pathogenic progression of MS and EAE. In light of the pharmacological profile of Scopoletin and its reported immunoregulatory properties, we hypothesized that Scopoletin might have a therapeutic effect on the animal model of MS and EAE. The purpose of this study was, therefore, to test the therapeutic activity of Scopoletin on EAE and to elucidate the underlying therapeutic mechanisms of its action, as part of our ongoing search for development of immunomodulation medicine extracted from medical plants or edible plants (Zhao et al., 2018).

## Materials and Methods

### Reagents and Animals

Scopoletin was purchased from Sigma (Sigma-Aldrich, St. Louis, MO). C57BL/6 mice, female, 8 weeks old, were obtained from the Fourth Military University (Xi’an, China). Studies were carried out by a protocol approved by the Animal Ethics Experimental Committee at of Shaanxi Normal University, and all experiments were operated strictly according to approved institutional guidelines and regulations.

### EAE Induction and Treatment

EAE was induced by subcutaneously injecting an emulsion containing 200 μg Myelin Oligodendrocyte Glycoprotein_35–55_ (MOG_35–55_) (Genescript, Piscataway, NJ), complete Freund’s adjuvant (CFA) (Sigma-Aldrich), and 5 mg/ml *Mycobacterium tuberculosis* H37Ra (Difco Laboratories, Lawrence, KS), and intraperitoneally (i.p.) injected 200 ng pertussis toxin (Sigma-Aldrich) in PBS on day 0 and 2 postimmunization (p.i.) (Zhang et al., 2015). Disease progression of EAE mouse was evaluated daily in a blind way by two researchers, scoring standard for EAE disease progression as follows: 0, no symptoms; 0.5, stiff tail; 1, limp tail; 1.5, limp tail and waddle; 2, paralysis of one limb; 2.5, paralysis of one limb and weakness of one other limb; 3, complete paralysis of two hind limbs; 4, moribund; and 5, death. (Li et al., 2016). Scopoletin was prepared in dimethylsulfoxide (DMSO) at 100 mg/ml for stock, which was further diluted 20 times with PBS as a working solution. DMSO (5%) was dissolved in PBS as vehicle. Vehicle or Scopoletin (50 mg/kg) was injected i.p. daily from the day 0 p.i.

### Histopathology

Mice were killed at day 20 postimmunization (p.i.). To assess CNS histopathological, mice were perfused through the heart with cold PBS; spinal cord was then fixed with 4% paraformaldehyde, cut into 7-μm sections, and stained with hematoxylin and eosin (H&E) or Luxol fast blue (LFB) for evaluation of inflammation or demyelination, respectively. Slides were evaluated in a blinded fashion for inflammation using a 0–3 scale as description, and two investigators selected 10 areas in the white matter of the spinal cord and scored for slides, evaluation methods, and criteria as description followed our previous studies (Li et al., 2016). The white matter area was manually outlined, and the Image-Pro Plus software was used to calculated areas (%) of demyelination for evaluation of demyelination (Li et al., 2017). Demyelination area percentage was quantified as the area without LFB staining (white) in white matter divided by the area white matter of spinal cord slices. Fixed spinal cords were embedded in optimum cutting temperature (OCT) solution (Tissue-Tek, Sakura Finetek, Japan) and then sectioned coronally cut into 12 μm. Finally, sections stained with primary and secondary antibodies. Results were acquired by Nikon Eclipse E600 fluorescent microscopy (Melville, NY).

### Mononuclear Cells (MNCs) and BMDCs Preparation

Spleens were mechanically grinded and filtrated with a 100-µm cell strainer (Falcon, Tewksbury, MA) to harvest splenic cells, subsequently incubated with red blood cell lysis buﬀer (Biolegend, San Diego, CA) for 60 s. After washing cells with cold PBS, cells were stimulated with MOG_35–55_ *in vitro*. To acquire CNS infiltrating cells, the protocol was followed as previously described (Li et al., 2017). To acquire the bone-marrow-derived dendritic cells (BM-DCs), femurs and tibias were separated from the naive adult C57BL/6 mice, and the cells were blown out of the bone marrow with a syringe full of the precooled PBS. After filtering with 100-µm cell strainer, the harvested cells were subsequently cultured with PRMI1640 containing granulocyte colony-stimulating factor (GM-CSF) cytokine (10 ng/ml) until the ninth day to obtain mature BM-DCs. Dendritic cells are cultured in medium without GM-CSF cytokine after ninth day.

### Drug Preparation for Cell Experiments

Stock solution of Scopoletin was dissolved into the concentration of 30 mM for cell experiments with DMSO.

### T-cell Proliferation Assay

Splenic CD4+ T cells were isolated from the naive adult C57BL/6 mice using a CD4+ T Cell Isolation Kit (Miltenyi Biotec) and labeled with CFSE. Both BM-DCs and T cells were cocultured at 1:10 ratio in the presence of 10 μg/ml of MOG_35–55_ (Genescript, Piscataway, NJ) and 100 ng/ml of lipopolysaccharide (LPS) (Sigma-Aldrich). T-cell proliferation was measured by analyzing CFSE intensity by flow cytometry after 72 h.

### Enzyme Linked Immunosorbent Assay (ELISA)

DCs culture medium or supernatants from splenocytes were harvested at 18 or 72 h, and sandwich ELISA was performed to determine the concentrations of cytokine production by ELISA kits (R&D Systems, Minneapolis, MN). according to the manufacturer’s instructions, including IFN-γ, IL-17, GM-CSF, IL-1β, IL-6, and IL-23. Briefly, the capture antibody was used to precoat overnight. Then, the plate was washed with wash buffer (R&D Systems, Minneapolis, MN). for three times. Blocking with 1% BSA in PBS for 1 h, gradient standard, and samples were added to the plate for 2 h incubation. Next, the samples and standard were removed clearly, and the detection antibody was incubated for 2 h. The wells were again washed, a TMB substrate solution was added to the wells, and color developed in proportion to the amounts of cytokines. The Stop Solution changed color from blue to yellow, and the intensity of the color was measured at 450 nm. A standard curve was run for each microwell plate. According to the standard curve, the concentration of cytokine was determined.

### Flow Cytometry Analysis

For surface-marker staining, cells were washed with PBS and incubated 30 min at 4°C with anti-CD4, anti-CD8, anti-CD11b, anti-CD11c, anti-CD80, and anti-CD86 (BD Biosciences, San Jose, CA) or isotype control Abs according to the manufacturer’s instructions to dilute. With 25 or 10 μg/ml MOG_35–55_ to stimulate splenocytes or infiltrating MNCs in CNS for 72 h or overnight to analyze Th1 and Th17 response to MOG_35–55_. For intracellular staining, cells were stimulated with 50 ng/ml PMA, 500 ng/ml ionomycin, and GolgiPlug for 5 h. For blocking the nonspecific staining, 1 μg Fc Block reagent for the FcγII/III receptor (BD Biosciences, San Jose, CA) was addded to 1 × 10^6^ cells in 100 μl staining buffer (BD Biosciences) and incubated for 15 min at 4°C. The cells are then washed. For surface staining, cells were incubated with anti-CD4, anti-CD8, anti-CD11b, anti-CD11c, anti-MHC class II, anti-CD80, and anti-CD86. Then, cells were fixed and permeabilized by Fix & Perm Medium (Invitrogen, Waltham, MA), using Abs anti-IL-17, anti-IFN-γ, or anti-GM-CSF (BD Biosciences, San Jose, CA) and were incubated overnight at 4°C to stain intracellular cytokines. FACS Aria was used for flow cytometric analysis (BD Biosciences, San Jose, CA). The results were evaluated by FlowJo (Treestar, Ashland, OR).

### Western Blot

Mature BM-DCs were placed in six-well plates with a density of 1.5 × 10^6^ cells/ml and stimulated with 100 ng/ml LPS to induce DC activation. Meanwhile, 100 μM Scopoletin was added to DCs. After 18 h, proteins were extracted from BM-DCs as described. The total protein contents were measured by Pierce^™^ BCA Protein Assay Kit (Thermo Fisher Scientific, Rockford, MA). DC proteins were equally loaded onto SDS-PAGE, electrophoresed, transferred onto PVDF membranes, and after blocking with 5% nonfat milk in TBS, the PVDF membranes were incubated with primary antibody over 12 h at 4°C (Li et al., 2018), including JNK, p-JNK, NF-κB, p-NF-κB, p38, p-p38 (cell signal technology), IRF-7, ERK1/2, p-ERK1/2 (Abcam, Cambridge, UK), and p-IRF-7 (Signalway Antibody, College Park, MD). After washing, the membranes were incubated with 0.5% horseradish peroxidase-labeled IgG. Temembrane-bound antibodies were detected with an Immolilon^™^ Western chemiluminescent HRP Substrate (Millipore, Billerica, MA) and analyzed with an ImageJ (ChemiDoc XRS System; Bio-Rad).

### Quantitative RT-PCR

Total RNA was extracted by RNeasy Mini Kit (Qiagen, Valencia, CA). cDNA was subsequently synthesized by QuantiTect^®^ Reverse Transcription Kit (QIAGEN). Quantitative RT-PCR was performed with the QuantiFast^™^ SYBR^®^ Green PCR Kit (QIAGEN) under standard thermocycler conditions (Applied Biosystems, Foster City, CA). Quantification was performed by normalization with housekeeping genes glyceraldehyde 3-phosphate dehydrogenase (GAPDH) and relative expression level as fold changes *via* the 2^−ΔΔCt^ method. Primers are summarized in **Supplementary Table 1**.

### Statistical Analysis

Results are described as mean ± SD. Statistical differences between two or multiple groups were implemented using Student’s *t*-test or the ANOVA. *P* < 0.05 was considered statistically significant. All statistical analyses were performed by GraphPad Prism 6 (GraphPad, La Jolla, CA).

## Results

### Scopoletin Suppressed the Development of EAE

**Figure 1:**
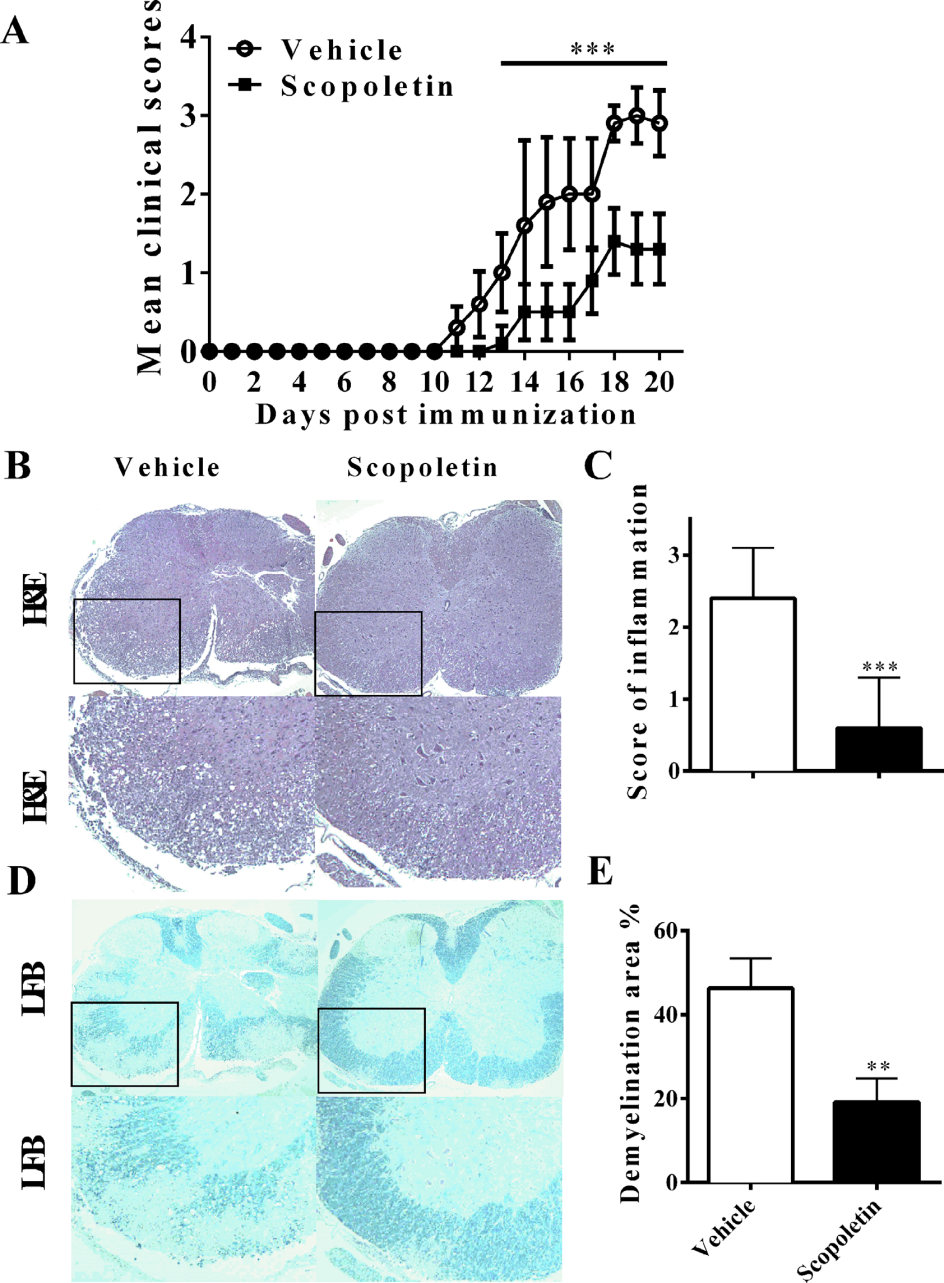
Scopoletin suppressed the development of experimental autoimmune encephalomyelitis (EAE). **(A)** Mice were injected i.p. with vehicle or Scopoletin (50 mg/kg) daily from day 0 p.i. to day 20 p.i. experimental autoimmune encephalomyelitis (EAE) development were evaluated and recorded daily following a 0–5 scale. **(B)** Hematoxylin and eosin (H&E), below is high magnification of H&E analysis of the spinal cord sections, and **(D)** Luxol fast blue staining, below is high magnification of Luxol fast blue (LFB) analysis of the spinal cord sections. **(C)** Mean score of inflammation in H&E staining. **(E)** Quantification of demyelination area was analyzed by Image-Pro Plus software. Scale bar = 100 µm. *n* = 8 mice each group. Symbols represent mean ± SD, ** *P* < 0.01 and *** *P* < 0.001, determined by two-way ANOVA **(A)**, or Student’s *t*-test **(B, D, F)**. Data are combined from three independent experiments.

Previous studies have shown that Scopoletin is safe at the dosage of 10–100 mg/kg daily with no other side effects in *in vivo* experiment (Jamuna et al., 2015; Basu et al., 2016; Zeng et al., 2017). To assess the effect of Scopoletin on the disease course of EAE, EAE mice were i.p. injected Scopoletin (50 mg/kg) from day 0 p.i. Vehicle-treated EAE mice demonstrated a progressive increase in disease severity after day 10 p.i., while the Scopoletin-treated group showed a marked decrease in mean disease course from day 14 to 20 p.i. (**Figure 1A**). Consistent with the disease development, mice treated with vehicle demonstrated abundant inflammatory cell infiltrated in the white matter compared with the Scopoletin-treated EAE mice (**Figures 1B, C**). At the same time, there was a dramatic decrease in demyelination areas in the spinal cord of Scopoletin-treated EAE mice compared to control (**Figures 1D, E**). In the therapeutic regimen, Scopoletin administration starting from disease onset (day 11 p.i.) effectively ameliorated EAE progression (**Supplementary Figure 1**). Together, these results indicate that Scopoletin effectively suppressed EAE pathogenesis.

### Scopoletin Treatment Inhibited DC Activation *In Vivo*

**Figure 2:**
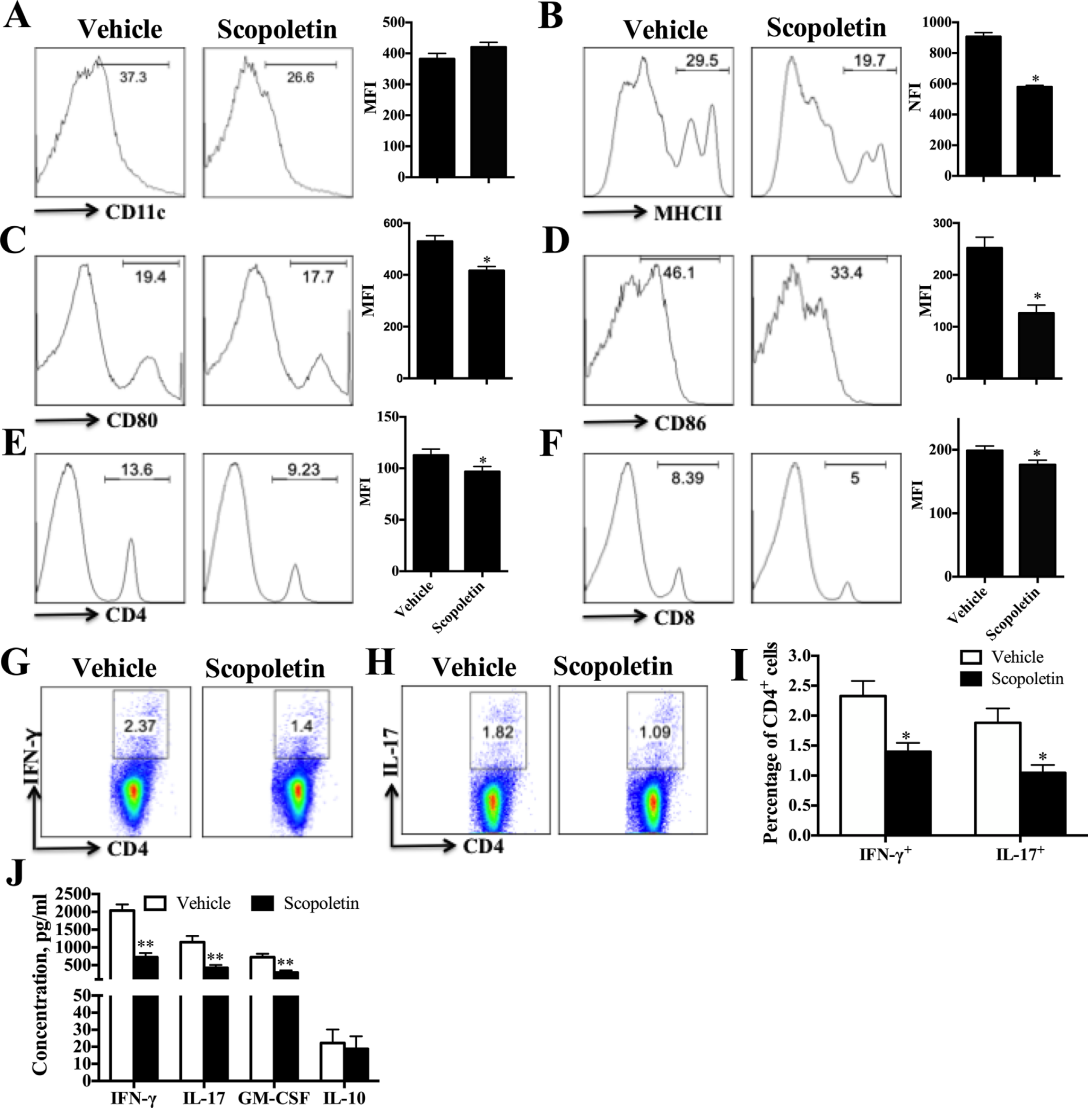
Scopoletin treatment reduced periphery inflammatory responses. Scopoletin- or vehicle-treated EAE mice were killed at day 20 p.i. and spleens were harvested. **(A–F)** Splenocytes were cultured in the presence of MOG_35–55_ (25 μg/ml) for 72 h and analyzed for the median fluorescence intensity (MFI) of **(A)** CD11c^+^, **(B)** MHC II^+^, **(C)** CD80^+^, **(D)** CD86^+^ (the positive cells of CD80 and CD86 are gated below the CD11c positive cells), **(E)** CD4^+^, and **(F)** CD8^+^. **(G**, **H)** Frequencies of Th1 and Th17 cells were assessed by flow cytometry. **(I)** Percentages of Th1 and Th17 cells were analyzed. **(J)** Supernatants derived from splenocyte cultures were analyzed for the level of indicated cytokines. Figures **(A–H)** are one representative of three independent experiments. Data **(A–F, I, J)** were determined by Student’s *t*-test and symbols represent mean ± SD, *P < 0.05 and **P < 0.01.

To assess the impact of Scopoletin on peripheral immune responses in EAE, splenocytes were harvested from EAE mice treated with Scopoletin or vehicle at day 20 p.i. and cultured in the presence of MOG_35–55_ (25 μg/ml) for 72 h. Expression of costimulatory molecules on CD11c^+^ DCs was examined by flow cytometry, which showed significantly decreased levels of MHC class II, CD80 and CD86 in Scopoletin-treated EAE mice compared to the vehicle group (**Figures 2A–D**). These results indicate that Scopoletin inhibited DC activation during the development of EAE.

Consistent with the observed effect of Scopoletin in EAE and DC activation, there was a remarkable diminishment in the percentages of CD4^+^ and CD8^+^ T cells in the periphery (**Figures 2E, F**). The percentages of Th1 and Th17 cells were also determined by intracellular staining and showed an obvious inhibition under Scopoletin treatment (**Figures 2G–I**). When stimulated with MOG_35–55_ *ex vivo*, splenocytes of Scopoletin-treated EAE mice secreted significantly lower levels of MOG-induced IFN-γ, IL-17, and GM-CSF (**Figure 2J**). When EAE symptoms is at its peak, spleen cells were harvested at 15 days p.i. and stimulated with MOG_35–55_ for 3 days. The concentration of IFN-γ, IL-17, and GM-CSF in cells culture supernatants were significantly decreased in Scopoletin-treated group (**Supplementary Figure 3A**). Furthermore, the inflammatory cytokine expression levels from sera were also detected by ELISA and RT-PCR. Although we did not detected protein expression by ELISA due to limited samples from sera, RT-PCR results showed that IL-17A, GM-CSF, and IL-1β expression were significantly decreased after Scopoletin treatment (**Supplementary Figure 3B**). These results indicated that Scopoletin alleviated the disease development of EAE by altering expression of costimulatory molecules and reduced activation of DCs, inhibiting Th1 and Th17 cells development, as well as suppressing proinflammatory cytokine secretion.

### Scopoletin Treatment Alleviated CNS Inflammation

**Figure 3:**
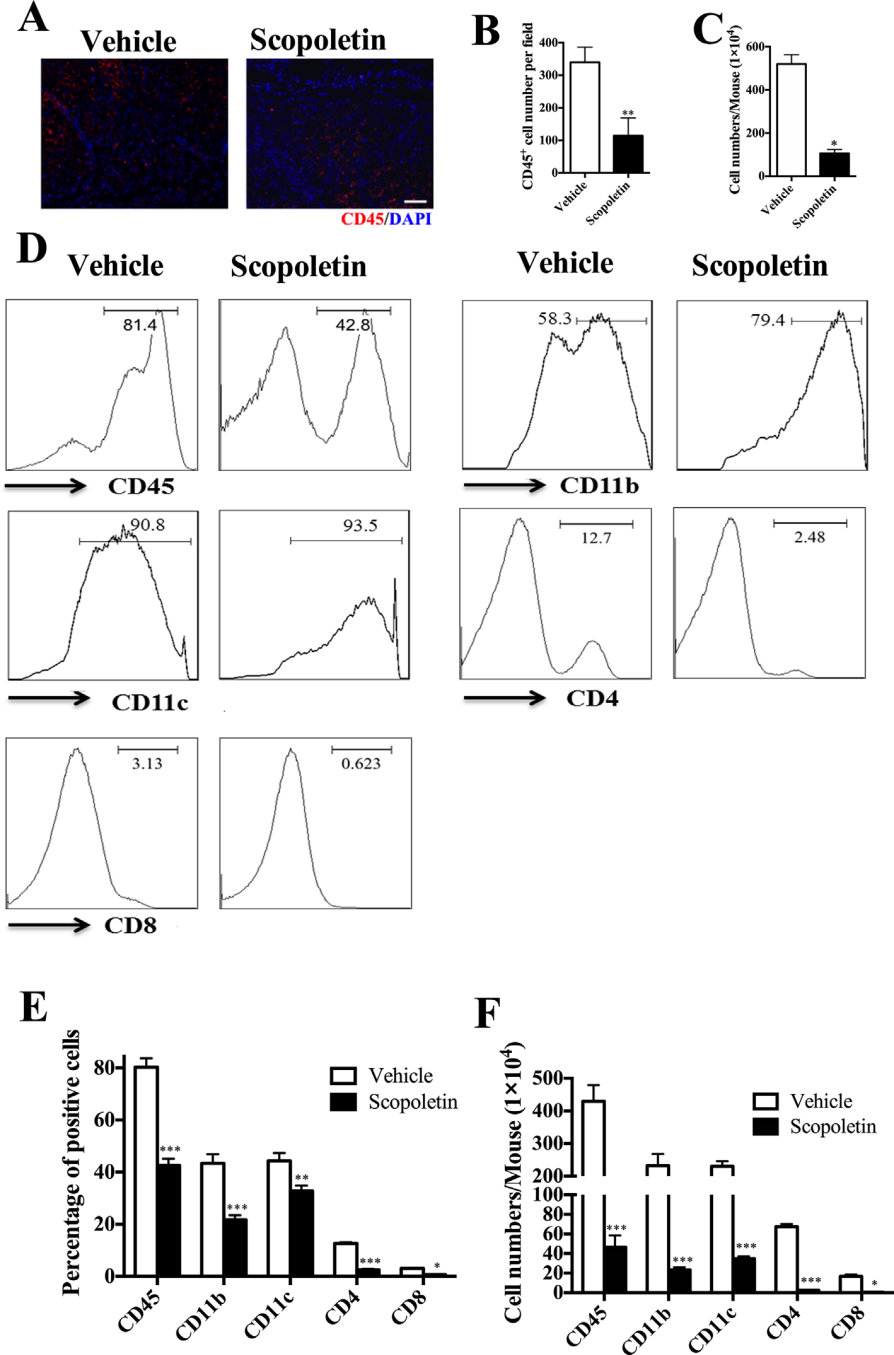
Scopoletin treatment alleviated central nervous system (CNS) inflammation. Scopoletin- or vehicle-treated EAE mice were killed at day 20 p.i., mononuclear cells (MNCs) were isolated from spinal cords and brains. **(A, B)** Spinal cords were subjected to immunofluorescent staining analysis. Representative sections of thoracic spinal cord from vehicle-treated mice or Scopoletin-treated mice were stained with CD45 to evaluate inflammatory infiltration. Scale bar = 100 µm. **(C)** Total MNCs numbers in CNS were counted under light microscopy. **(D, E)** The percentages of CD45^+^ leukocytes, CD11b^+^ microglia/macrophage cells, CD11c^+^ DCs, CD4^+^, and CD8^+^ T cells were measured by flow cytometry. **(F)** Absolute numbers of different subtypes of CNS infiltrates were calculated by multiplying the percentages of these cells with total numbers of MNCs in each spinal cord and brain tissue. Figures **(A, D)** are one representative of three independent experiments. Data were determined by Student’s *t*-test, and symbols represent mean ± SD, *P < 0.05, **P < 0.01, and ***P < 0.001.

In the CNS, effector T cells produce a number of molecules that recruit inflammatory leukocytes, ultimately leading to demyelination (Thome et al., 2014b). To evaluate the effect of Scopoletin on EAE-related CNS pathology, thoracic spinal cords were harvested from Scopoletin- or vehicle-treated EAE mice. Immunofluorescence evaluation of CNS tissues showed that Scopoletin markedly decreased infiltration of CD45^+^ cells compared with control mice (**Figures 3A, B**). The number of MNCs in the CNS of Scopoletin-treated mice was dramatically decreased compared to the vehicle group (**Figure 3C**). Furthermore, flow cytometry was used to analyze CD45^+^ (leukocyte), CD11c^+^ (DC), and CD11b^+^ (macrophage/microglia) cell populations. Results showed that the percentages and absolute numbers of these cells were significantly reduced in the CNS of Scopoletin-treated mice compared with vehicle-treated EAE mice. In addition, Scopoletin-treated mice have lower percentages and absolute numbers of CD4^+^ and CD8^+^ T cells in the CNS (**Figures 3D–F**).

**Figure 4:**
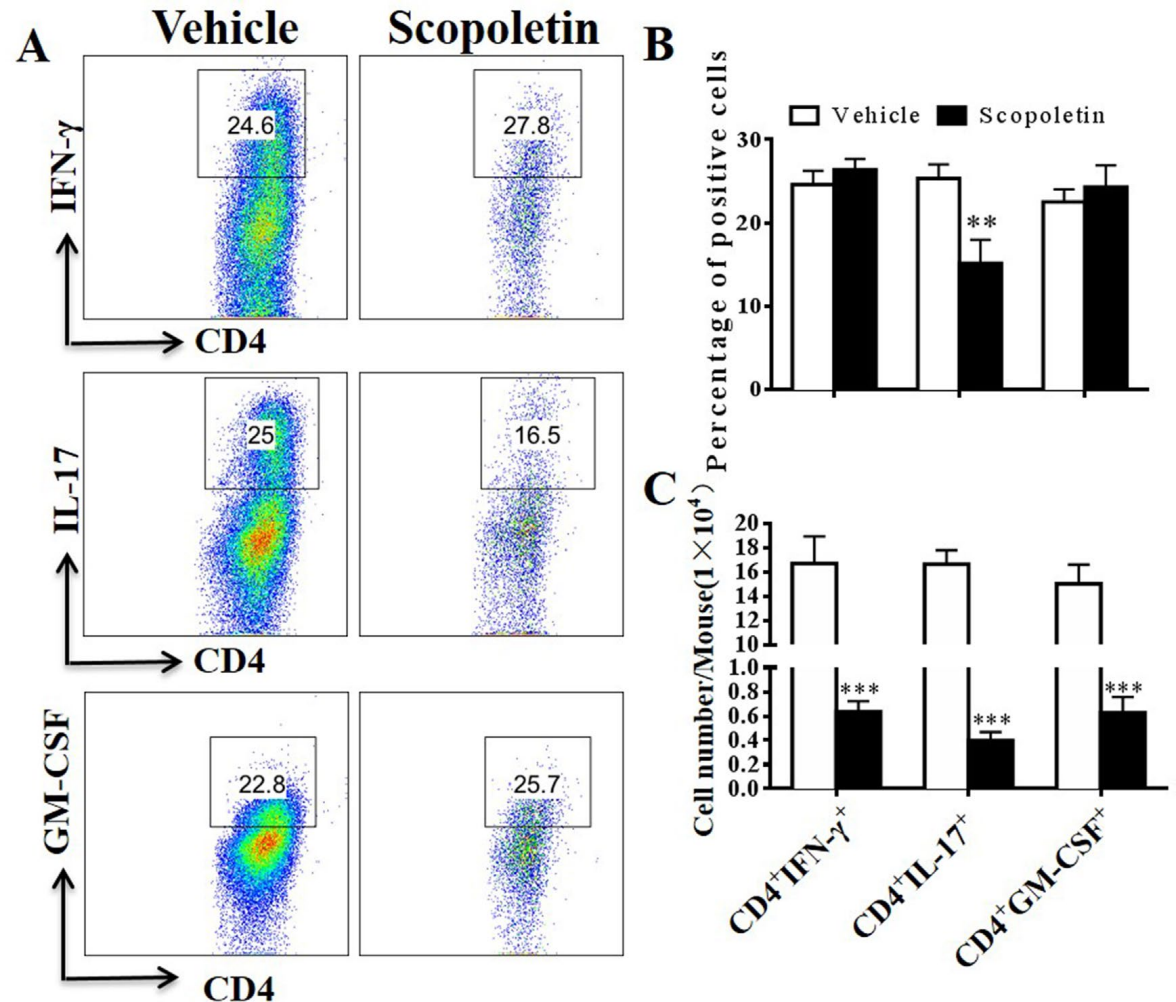
Scopoletin treatment reduced numbers of IFN-γ, IL-17-, and GM-CSF-producing CD4^+^ T cells in the CNS. Scopoletin- or vehicle-treated EAE mice were killed at day 20 p.i., spinal cords and brains were harvested, and MNCs were isolated. **(A, B)** Percentages of IFN-γ-, IL-17-, and GM-CSF-producing CD4^+^ T cells were assessed by flow cytometry. **(C)** Absolute numbers of these cells were calculated by multiplying their percentages of cytokine-producing cells and total CD4^+^ cell numbers in each CNS. Data are representative of three independent experiments. Symbols represent mean ± SD (n = 5 each group), **P < 0.01 and ***P < 0.001, determined by Student’s t-test.

Emerging data suggest that both interferon-γ-producing (Th1) and interlukin-17-producing Th17 (Th17) cells contribute to CNS autoimmunity and mediate disease pathogenesis in EAE (El-Behi et al., 2010). In addition, although various cell types produce GM-CSF, myelin-specific CD4+ T cells are essential to EAE development (Rasouli et al., 2015). To explore potential mechanisms underlying the therapeutic effect of Scopoletin in EAE, we measured the numbers of Th1, Th17, and pathogenic CD4^+^GM-CSF^+^ (Rasouli et al., 2015) cells in the CNS of EAE mice. As shown in **Figures 4A–C**, the percentages and absolute numbers of Th17 cells were reduced in the Scopoletin-treated group in comparison to the vehicle-treated mice. While the percentages of CD4^+^IFN-γ^+^ and CD4^+^IL-17^+^ T cells were not significantly changed, the absolute numbers of these cells were decreased in the Scopoletin-treated group (**Figures 4A–C**) due to the reduced total numbers of CD4^+^ T cells in the CNS after Scopoletin treatment (**Figures 3D–F**). CD4^+^GM-CSF^+^ T cells infiltration in the CNS was found in these groups. Our data therefore suggest that the effect of Scopoletin in alleviating EAE development may be due to an alteration in the differentiation of Th1, Th17, and CD4^+^GM-CSF^+^ T cells (**Figures 4A–C**).

### Scopoletin Inhibited the Activation of Murine BM-DCs *In Vitro*

**Figure 5:**
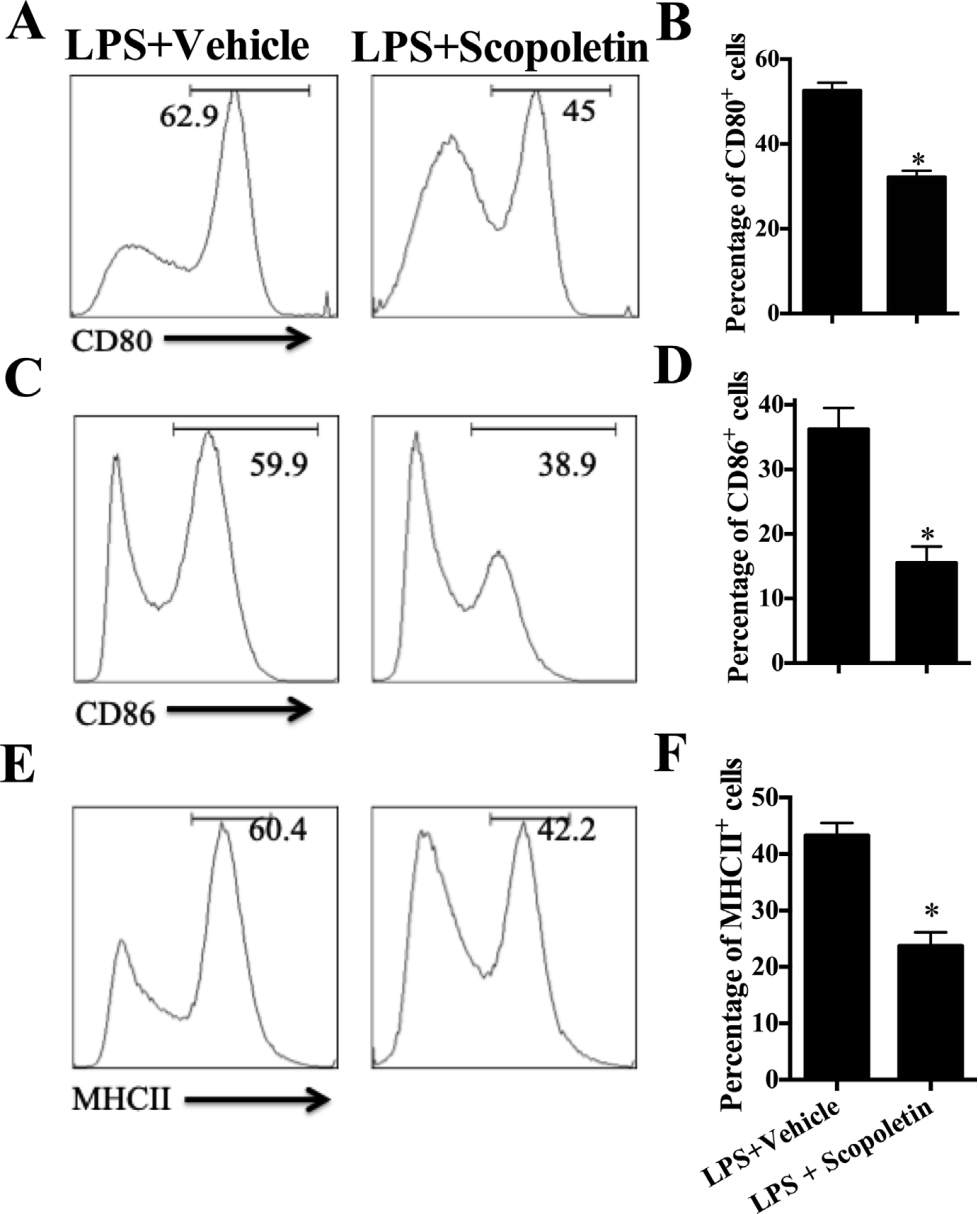
Scopoletin inhibited the activation of murine bone marrow-derived dendritic cells (BM-DCs) by inhibiting NF-κB signaling *in vitro*. BM-DCs were generated and stimulated with 100 ng/µl LPS, and cultured with Scopoletin at a dose of 100 µM. After 18 h, expression of **(A)** CD80, **(C)** CD86, **(E)** MHC II (the positive cells of CD80, CD86, and MHC II are gated below the CD11c positive cells) was measured by flow cytometry following overnight incubation and treatment with or without Scopoletin. **(B, D, F)** Percentages of each molecule were counted. Figures **(A, C, E**) are one representative of three independent experiments. Statistical data are expressed as mean ± SD of three independent experiments. *P < 0.05 by Student’s *t*-test.

We have shown the inhibitory effect of Scopoletin on DC activation *in vivo* in EAE mice (**Figures 2A–D**); here, we evaluated its direct effects on DCs *in vitro*. To that end, BM-DCs were generated. Upon LPS stimulation, DCs expressed relatively high levels of MHC class II, CD80, and CD86, the markers of DCs activation, and their expression was significantly inhibited by Scopoletin treatment (**Figures 5A–F**). Scopoletin, thus, showed a potential for restricting DCs activation and inhibiting expression of costimulatory molecules in BM-DCs cultures.

**Figure 6:**
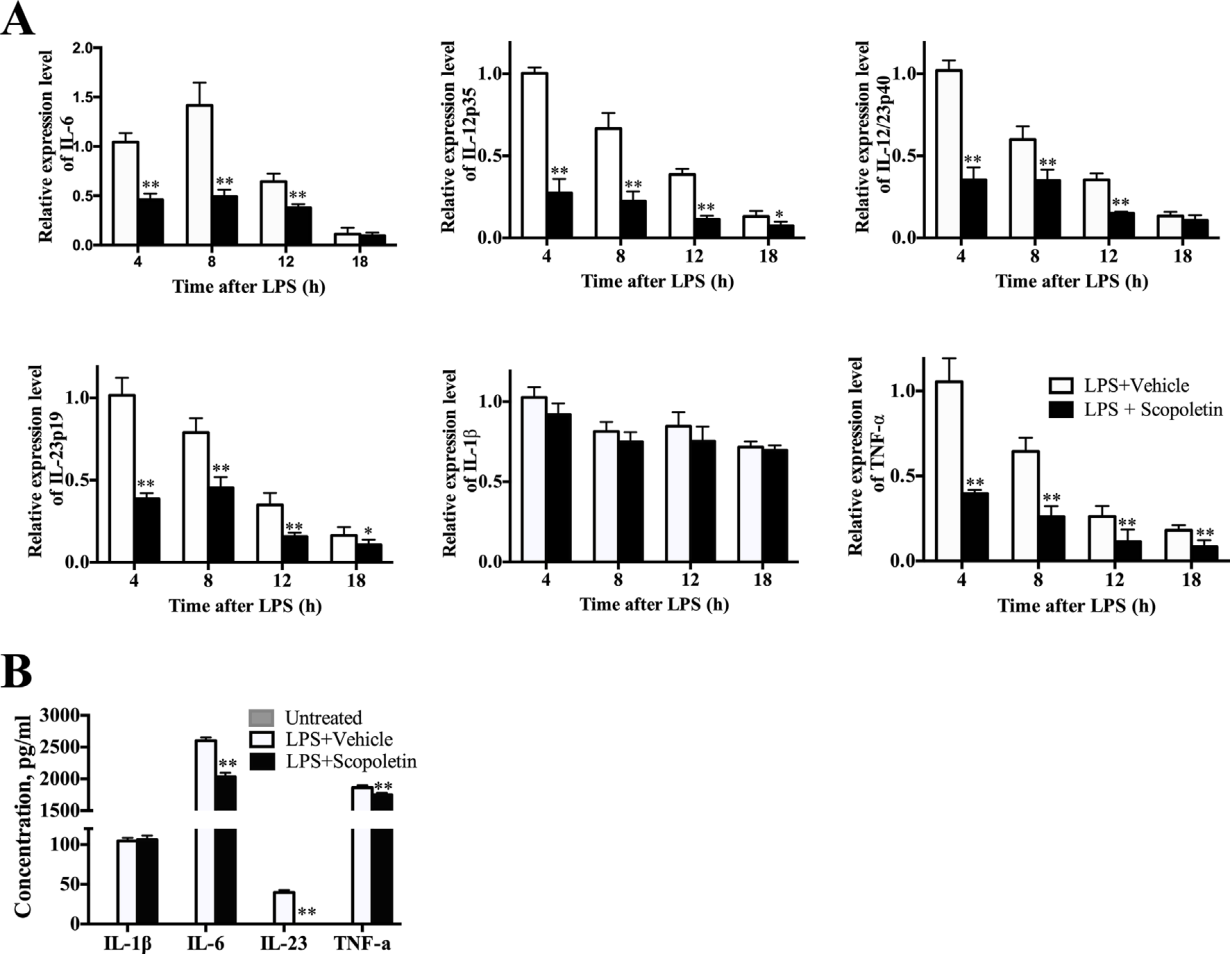
Scopoletin treatment decreased expression of inflammation-related genes and production of proinflammatory cytokines of BM-DCs. **(A)** BM-DCs were generated and activated with 100 ng/μl LPS. mRNA levels of inflammatory-related genes from BM-DCs were determined at 4, 8, 12, and 18 h by qRT-PCR after Scopoletin treatment. **(B)** Supernatants were assayed by ELISA for production of IL-1β, IL-6, IL-23, and TNF-α. Data are mean ± SD of three independent experiments. * *P* < 0.05 and ** *P* < 0.01 by two-way ANOVA.

To further examine the inhibitory effect of Scopoletin on BM-DCs activation, we then determined the mRNA levels of multiple inflammatory-associated genes expressed by BM-DCs, such as IL-6, IL-12p35, IL-12/IL23p40, IL-23p19, IL-1β, and TNF-α. Previous studies have shown that IL-12 is probably related to Th1 cells differentiation, and IL-23 and IL-6 are crucial to the Th17 cells differentiation (Luckheeram et al., 2012). Importantly, Scopoletin treatment caused a remarkably inhibition in the expression of these genes except for IL-1β (**Figure 6A**). ELISA analysis indicated the suppressive effects of Scopoletin on the secretion of IL-6, IL-23, and TNF-α by BM-DCs (**Figure 6B**). These findings suggest that the effect of Scopoletin treatment may be through inhibiting production of proinflammatory cytokines and expression of costimulatory molecules in DCs.

To test the effects of Scopoletin in DC-induced T-cell proliferation assays, we performed the DC and T-cell coculture and quantified the T-cell proliferation with CFSE; the results showed that Scopoletin reduced the T-cell proliferation *via* DCs induction (**Supplementary Figure 3C**).

### Scopoletin Inhibited NF-κB Signaling During LPS-Stimulated DC Activation *In Vitro*

**Figure 7:**
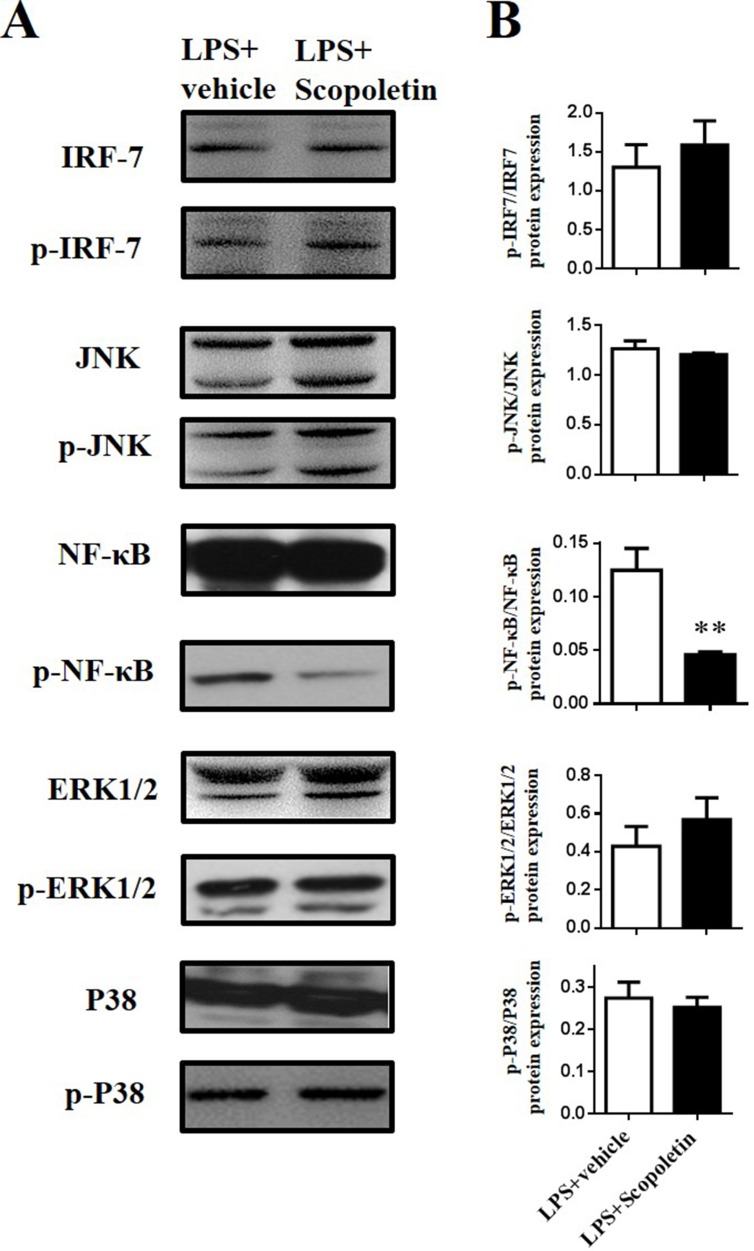
Scopoletin inhibited NF-κB signaling in LPS-stimulated BM-DCs *in vitro*. BM-DCs were activated with LPS and cultured with Scopoletin at a dose of 100 µM. After 18 h, cells were harvested and homogenized for Western blot. **(A)** Protein expression levels of nuclear transcription factors and protein kinase were measured by Western blot. **(B)** Densitometry was determined by ImageJ, and data were analyzed by GraphPad Prism 6. Data are expressed as mean ± SD of three biological replicates. **P < 0.01 by Student’s *t*-test. Figure A is one representative of three independent experiments.

It is well known that NF-κB and MAPK signaling pathways play a central role in the coordinated regulation of gene expression during DC activation (Lu et al., 2014; Baratin et al., 2015). Thus, expression of the total and phosphorylation forms of IRF-7, p38, JNK, and NF-κB in BM-DCs was determined by Western blot. The level of phosphorylation of NF-κB was significantly lower in Scopoletin-treated BM-DCs when compared to DCs stimulated with LPS alone. In contrast, expression of IRF-7, p38, and JNK were not markedly affected by Scopoletin treatment (**Figures 7A, B**). In addition, BM-DCs were pretreated with SN50 (20 μM) for 1 h, a specific NF-κB peptide inhibitor (Lin et al., 1995), before LPS (100 ng/ml) stimulation and Scopoletin (100 μM) treatment, after 4 h cells were collected, and then, expression level of cytokines were determined by quantitative real time PCR (qRT-PCR). The expression of NF-κB downstream genes such as IL-1β and TNF-α was significantly decreased by Scopoletin and SN50 treatment. When combined with SN-50 and Scopoletin, TNF-α expression level was significantly enhanced compared with Scopoletin only (**Supplementary Figure 3D**). These data suggest that Scopoletin may exert its action and ameliorate the progression of EAE specifically through inhibiting NF-κB signaling.

## Discussion

In this study, we provide evidence, for the first time, of the anti-inflammatory activities and protective effects of Scopoletin in the mouse model of MS, EAE. Our data present a promising bioactive drug, derived from a natural source, for the treatment of MS and possibly other autoimmune diseases.

In addition, Scopoletin is also the main coumarin constituent occurring in the stems of *Erycibe obtusifolia*, a classical medicinal plant that has demonstrated various biological activities mainly used for rheumatoid arthritis with a long history (Pan et al., 2010). Scopoletin has been reported to have anti-inflammatory properties, e.g., inhibition of lymphocyte proliferation and reduction of cytokine production (Arcos et al., 2006). *In vitro* studies showed that Scopoletin inhibited proinflammatory cytokine secretion from RAW 246.7 and HMC-1 cell lines (Moon et al., 2007; Connell et al., 2017). Scopoletin also exhibited anti-inflammatory, antioxidant, and antiacetylcholinesterase potential against Alzheimer’s disease (Garcia-Morales et al., 2015). However, its role is unknown in the regulation of APC functions in an autoimmune disease such as MS.

In autoimmune diseases, DCs as professional APCs have a potent ability to trigger naive T-cell reaction and activate an autoreactive response (Thompson et al., 2018), and decreasing CD80/86 prevented T-cell activation and Th-cell differentiation by inhibiting the costimulatory second signal (Grimbert et al., 2011; Yamamoto et al., 2012). Our results showed that the therapeutic effects of Scopoletin were largely dependent on the suppression of DC activation. Scopoletin reduced the expression of costimulatory molecules CD80 and CD86 as well as MHC class II *in vivo* and *in vitro*. Furthermore, in MS patients and EAE mice, there is a marked increase in the levels of IFN-γ and IL-17, signature cytokines for Th1 and Th17 cells, respectively (Arellano et al., 2017). As drivers of autoimmune response, pathogenic Th1 and Th17 cells are therefore considered to be the main culprits in MS and EAE (Grigoriadis et al., 2015). Our data demonstrated that Scopoletin treatment remarkably decreased the percentages of Th1 and Th17 cells. On the other hand, GM-CSF, which can be produced by both Th1 and Th17 cells, is required for encephalitogenicity of these pathogenic T-cell subsets (Codarri et al., 2011; El-Behi et al., 2011). Reduced numbers of CD4^+^GM-CSF^+^ T cells in the spleen and CNS following Scopoletin treatment would, therefore, be an important mechanism underlying the effect of Scopoletin on EAE. When the direct effect of Scopoletin on Th1 and Th17 cell differentiation was further investigated *in vitro*, our results showed that diﬀerentiation of Th1 and Th17 was not significantly altered by Scopoletin treatment (**Supplementary Figure 2**). Furthermore, the inhibitory effect of Scopoletin treatment on Th1/Th17 cells *in vivo* in EAE mice must therefore be through an indirect mechanism, i.e., by inhibition of DC activation during EAE progression.

It has been well documented that IL-6 plays a key role in determination of Th17 polarization, and knockout of these cytokines completely inhibits EAE development (Giralt et al., 2013). In addition, IL-23 is important for expanding and maintaining the developing Th17 population (Smith and Colbert, 2014) and is required for differentiation of Th17 cells and the pathogenic role of these T cells (El-Behi et al., 2011). Given that DCs are major producers of these cytokines, differentiation of naive T cells into Th17 phenotype is dependent on activated DCs (Agalioti et al., 2018). IL-6 signaling plays a key role in the differentiation of CD4 + T cells, in combination with TGF-β, IL-1β, IL-23, and IL-6, promotes the differentiation of Th17 cells by activating transcription factors containing signal transducers and activators of transcription 3 (STAT3), retinoic acid-related orphan receptors gamma t (RORγt) (Yao et al., 2014). Here, we found that Scopoletin treatment significantly decreased expression of activation and costimulation markers of DCs and their IL-6 and IL-23 production, further indicating that Scopoletin blocked DC secretion of Th17-inducing cytokines, thus resulting in inhibited Th17 phenotype formation.

When antigen presentation occurs, epitope-major histocompatibility complexes identified by T-cell receptors and costimulatory signals are sent by interaction of CD80, CD86 of DCs and CD28 expressing on T cells, as well as the cytokine milieu formed by DCs and other inflammatory cells in the microenvironment (Thome et al., 2014b). These signals working together results in T cell polarization into Th1, Th17, and other effector CD4^+^ T cells. Studies have shown that the activation of DCs is possibly regulated by three intracellular signaling pathways, including NF-κB, MAPKs, and PI3K (Chesi et al., 2016; Rescigno et al., 1998). To further explore the mechanism of Scopoletin-induced amelioration of EAE, we investigated the effects of Scopoletin on BM-DCs *in vitro,* which indicated decreased expression of a series of inflammation-related genes. While the activation of MAPKs containing ERK, p38, and JNK was not prominently influenced in LPS-stimulated DCs, Scopoletin treatment exerted a profound inhibitory effect on NF-κB activity during DC activation. It was shown that Scopoletin markedly suppressed the phosphorylation of NF-κB, which plays a significant role in the activation and maturation of DCs. Previous studies showed that IL-6 gene expression was reduced through downregulating of NF-κB signaling (Chiang et al., 2014), and the promoter activity of IL-23 was modulated by the NF-κB pathway. Our results indicated that the therapeutic effect of Scopoletin may be mediated by inhibiting the NF-κB signaling pathway, which is vitally involved in producing proinflammatory cytokines.

In summary, we reported, for the first time, that natural compound Scopoletin ameliorated the severity of EAE by regulating DC activation and reducing CNS inflammation *via* suppression of NF-κB signaling. Scopoletin treatment could therefore be a potential candidate for the modulation of inflammatory conditions of autoimmune diseases.

## Conclusion

Our study demonstrates that natural products Scopoletin derived from medical or edible plants will be valuable in developing a novel therapeutic agent for MS in the future.

## Data Availability

The raw data supporting the conclusions of this manuscript will be made available by the authors, without undue reservation, to any qualified researcher.

## Ethics Statement

This study was carried out in accordance with the recommendations of ‘institutional guidelines and regulations approved by the Animal Ethics Experimental Committee at of Shaanxi Normal University’, ‘the Animal Ethics Experimental Committee at of Shaanxi Normal University’. The protocol was approved by the ‘Animal Ethics Experimental Committee at of Shaanxi Normal University’.

## Author Contributions

FZ, YZ, TY, and XL conceived and designed the experiments. FZ, Z-QY, TY, H-RF, and J-JH carried out the experiments. FZ, YZ, TY, JT, and XL analyzed data and wrote the manuscript. Z-ZW and XL cosupervised the study and revised the paper. All authors read and approved the final manuscript.

## Funding

This study was supported by the Chinese National Natural Science Foundation (Grant No. 81771345, 81501062, 31670299), the Natural Science Foundation of Shaanxi Province, China (Grant No. 2018JZ3001, 2018JQ8033, 2019KJXX-022), and the Fundamental Research Funds for the Central Universities (Grant No. GK201903062, GK20182010, GK201701009, 2018CSLZ018, 2018CSLZ019, 2018CSLZ020, 201810718052).

## Conflict of Interest Statement

The authors declare that the research was conducted in the absence of any commercial or financial relationships that could be construed as a potential conflict of interest.
