# Corrigendum: Scopoletin Suppresses Activation of Dendritic Cells and Pathogenesis of Experimental Autoimmune Encephalomyelitis by Inhibiting NF-κB Signaling

**DOI:** 10.3389/fphar.2019.01037

**Published:** 2019-09-13

**Authors:** Fei Zhang, Yuan Zhang, Ting Yang, Ze-Qing Ye, Jing Tian, Hai-Rong Fang, Juan-Juan Han, Zhe-Zhi Wang, Xing Li

**Affiliations:** National Engineering Laboratory for Resource Development of Endangered Crude Drugs in Northwest China, The Key Laboratory of Medicinal Resources and Natural Pharmaceutical Chemistry, The Ministry of Education, College of Life Sciences, Shaanxi Normal University, Xi’an, China

**Keywords:** scopoletin, experimental autoimmune encephalomyelitis, multiple sclerosis, dendritic cells, NF-κB signaling

In the published article, author Zhe-Zhi Wang’s identity was incorrect. Instead of “co-author,” it should be “co-corresponding author.” The authors apologize for this error and state that this does not change the scientific conclusions of the article in any way. The original article has been updated.

