# Supplementary material for: Scopoletin Suppresses Activation of Dendritic Cells and Pathogenesis of Experimental Autoimmune Encephalomyelitis by Inhibiting NF-κB Signaling
Source: Front Pharmacol. 2019 Aug 2;10:863. doi: 10.3389/fphar.2019.00863 (PMC6688631; doi:10.3389/fphar.2019.00863)
Supplement: Supplementary file 1 [file DataSheet_1.docx]

**Sup Fig 1.** EAE mice were injected i.p. with vehicle or Scop (50 mg/kg) daily after the disease induction (day 11 p.i.). Clinical scores were recorded daily following a 0-5 scale. n = 5 mice each group. Symbols represent mean ± SD, ** *P* < 0.01, determined by two-way ANOVA. Data represent three independent experiments.


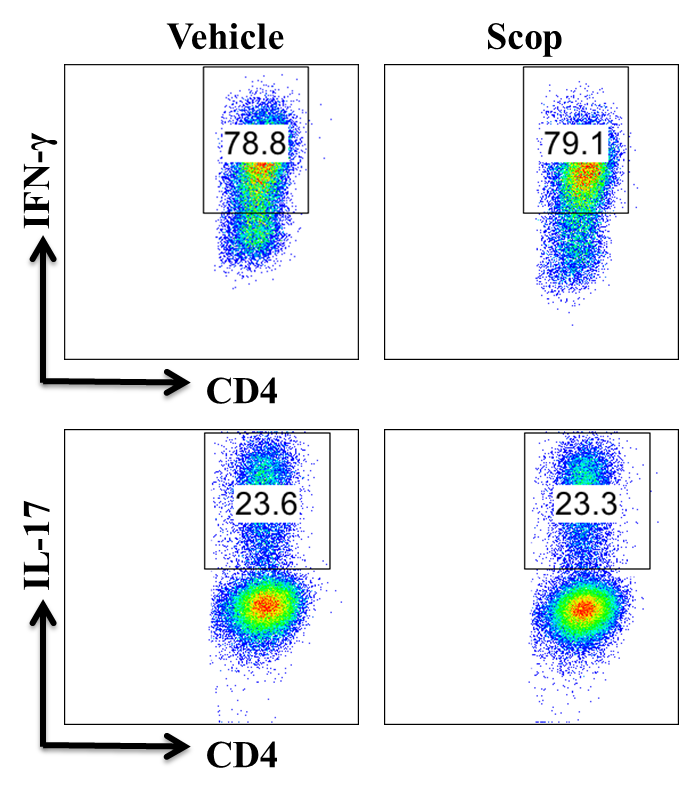


**Sup Fig 2.** Naïve CD4^+^ cells were cultured with Scop (100 μM) under the Th1 (IL-12, 5 ng/ml) and Th17 (TGF-β1, 2 ng/ml, IL-6, 20 ng/ml, IL-1β, 10 ng/ml, anti-IL-4, 10 μg/ml, and anti-IFN-γ, 10 μg/ml) polarizing condition for 3 days of culture. Percentage of Th1 and Th17 cells was analyzed by intracellular staining of IFN-γ and IL-17. Figures are representative of 3 independent experiments.





**Sup Fig 3.** A, Scopoletin- or vehicle-treated EAE mice were sacrificed at day 15 p.i. and splenocytes were isolated from spleens. Splenocytes were cultured in the presence of MOG_35–55_ (25 μg/ml) for 72 h and supernatants were analyzed for the level of indicated cytokines (mean ± SD, n = 5 each group). B, Scopoletin- or vehicle-treated EAE mice were sacrificed at day 12 p.i. and the sera samples were harvested. The expression of the cytokines of IL-17a, IL-17f, IFN-γ, GM-CSF, IL-1β and IL-6 as measured by RT-PCR. C, BM-DCs were pretreated with LPS (100ng/ml) and MOG_35–55_ (10 μg/ml) for 18 h, then aspirated supernatant and co-cultured with CFSE stained T cells at a 1:10 ratio. T cell proliferation was determined by flow cytometry at day 3 and quantified. C, BM-DCs were pretreatment with or without Sn50 (20 μM) for 1h, then added LPS (100 ng/ml) and Scopoletin (100 μM) for 4h. mRNA levels of IL-1β and TNF-α from BM-DCs were analyzed by real-time PCR. Symbols represent mean ± SD, * *P* < 0.5, ***P* < 0.01, determined by student’s t-test.


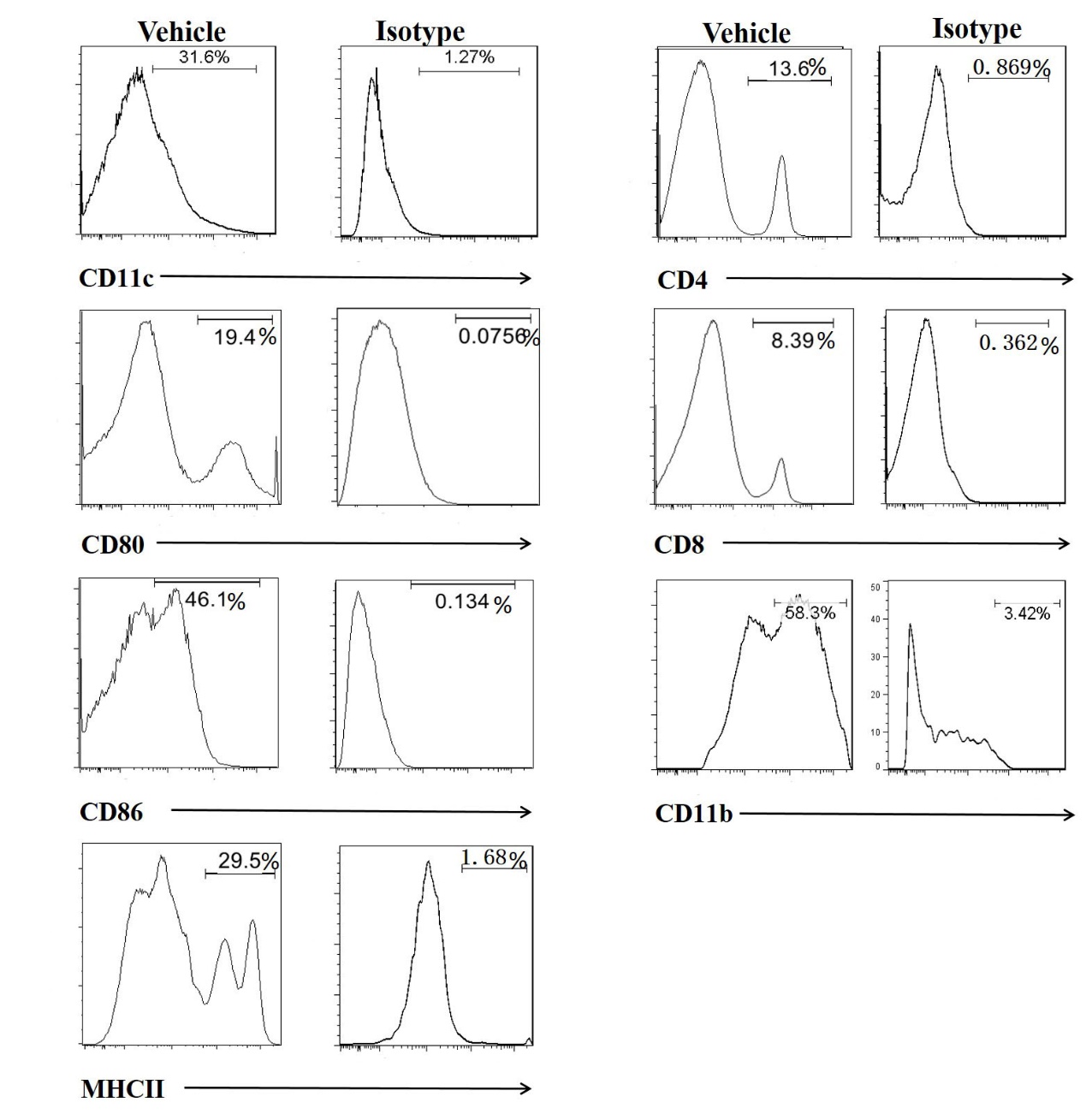


**Sup Fig 4** Cells were stained by Abs to CD11c, CD80, CD86, MHCII, CD4, CD8, CD11b surface-marker or isotype control Abs for 30 min on ice. Negative cells were excluded by isotype control and every cell subset was analyzed by FlowJo software.

**Supplementary table 1**

**Primers used for real-time quantitative RT-PCR analysis**

| Gene | Primers |
| --- | --- |
| GAPDH F | CCAATGTGTCCGTCGTGGATCT |
| GAPDH R | GTTGAAGTCGCAGGAGACAACC |
| IL-6 RT F | ACACATGTTCTCTGGGAAATCGT |
| IL-6 RT R | AAGTGCATCATCGTTGTTCATACA |
| IL-12p35 RT F | CATCGATGAGCTGATGCAGT |
| IL-12p35 RT R | CAGATAGCCCATCACCCTGT |
| IL-12p40 RT F | AGGTCACACTGGACCAAAGG |
| IL-12p40 RT R | TGGTTTGATGATGTCCCTGA |
| IL-23p19 RT F | GACTCAGCCAACTCCTCCAG |
| IL-23p19 RT R | GGCACTAAGGGCTCAGTCAG |
| IL-1β RT F | CTCTCCACCTCAATGGACAGA |
| IL-1β RT R | TGCTTGGGATCCACACTCTC |
| TNF-α RT F | GACGTGGAACTGGCAGAAGAG |
| TNF-α RT R | GCCACAAGCAGGAATGAGAAG |
